# Isolated Unilateral Hypoglossal Nerve Palsy Caused by Internal Carotid Artery Loop

**DOI:** 10.7759/cureus.14819

**Published:** 2021-05-03

**Authors:** Murat Dokdok, Selçuk Göçmen, Serdar Kahraman, Yaşar Kütükçü

**Affiliations:** 1 Radiology Department, Anadolu Medical Center, Kocaeli, TUR; 2 Neurosurgery Department, Anadolu Medical Center, Kocaeli, TUR; 3 Neurology Department, Anadolu Medical Center, Kocaeli, TUR

**Keywords:** carotid artery dissection, isolated hypoglossal nerve palsy, vascular compression, internal carotid artery loop, cerebral angiography

## Abstract

Isolated unilateral hypoglossal nerve (HN) palsy caused by vascular compression is a rare condition. We report a case of a 42-year-old male, presenting with tongue paresis and unilateral atrophy of the tongue due to an internal carotid artery (ICA) loop. The compression of HN by ICA loop and concomitant wall irregularities of the loop segment were observed in magnetic resonance imaging and digital subtraction angiography (DSA). The patient was managed with antithrombotic without the need of any further intervention. To our best knowledge, this is the first reported case of isolated compressive neuropathy of the HN caused by loop of the ICA. Here, the clinical presentation, etiology, and management of isolated HN palsy caused by vascular lesions are discussed along with the relevant literature.

## Introduction

As a rare clinical entity, isolated unilateral hypoglossal nerve (HN) palsy may result from a number of different pathologies. It is crucial to define the underlying pathology to manage the condition wisely. In literature, assorted arterial or venous vascular causes including internal carotid artery (ICA) dissection, vertebral artery (VA) compression, posterior inferior cerebellar artery (PICA) aneurysm, enlarged emissary vein, dural arteriovenous (AV) fistula and persistent hypoglossal artery were reported [1-3]. However, ICA loop related isolated cranial nerve palsy was not reported previously. We present here a case of ICA loop and concomittant dissection which caused isolated HN palsy.

## Case presentation

**Figure 1:**
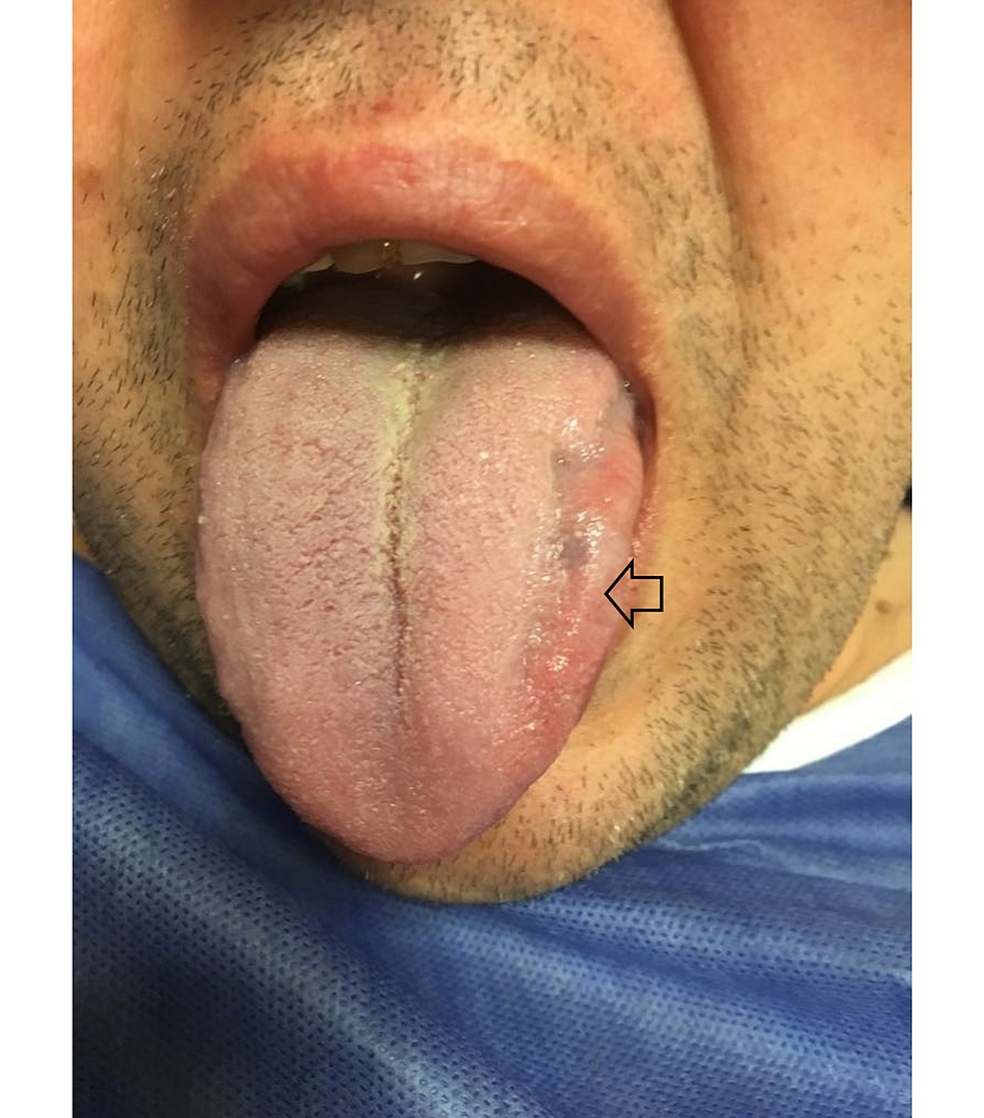
Atrophy on the left side of the tongue (open arrow) that deviated toward the paralyzed site

A 42-year-old male, who had been suffering from dysarthria and weakness of the left side of the tongue, over the past three months, was referred to our hospital. The patient’s history did not include any other significant trauma, infection, and hypertension, or other systemic diseases. Neurological examination revealed palsy of the HN and atrophy of the tongue on the left side (Figure 1). Neither tongue fasciculation nor any other neurological deficit was observed. The patient’s articulation and eating were mildly impaired as his symptoms gradually improved during the last couple of weeks. Needle electromyography demonstrated active denervation of the left HN with partial axonal degeneration. Other laboratory tests including blood tests were unremarkable.

**Figure 2:**
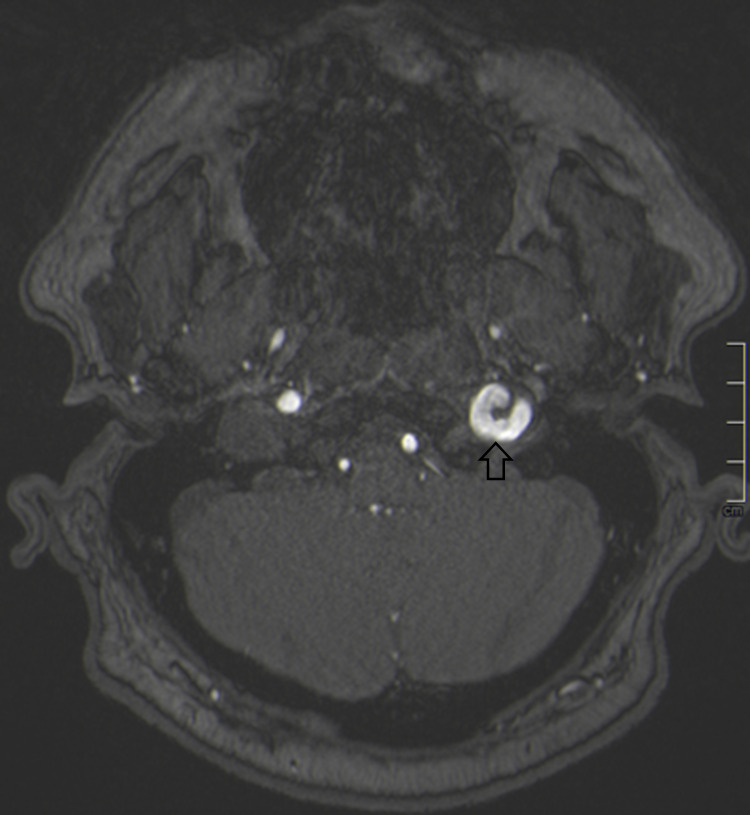
The axial raw image of time-of-flight magnetic resonance angiography showing vascular loop (open arrow) in the outlet portion of left hypoglossal canal

**Figure 3:**
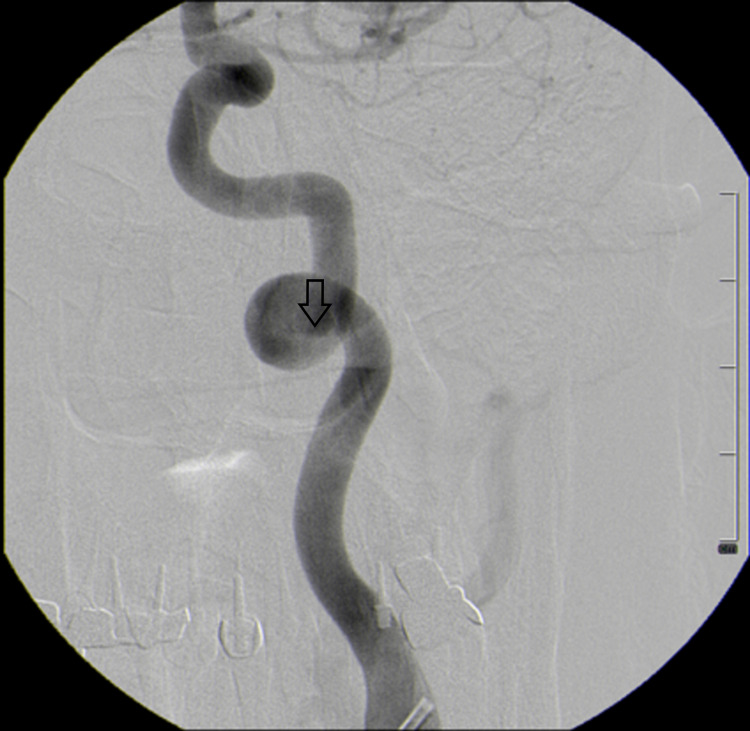
Digital subtraction angiography displaying type 3 loop of the internal carotid artery with wall irregularities (open arrow)

Magnetic resonance angiography (MRA) revealed a dilated vascular loop of the left ICA that obstructed the outlet portion of the left hypoglossal canal compressing the HN (Figure 2). Digital subtraction angiography (DSA) confirmed the dilatation of the distal cervical segment of the left ICA with type-3 loop and coiling (Figure 3). Furthermore, the inferior posterior region of the loop had prominent wall irregularities which might be consistent with late findings of ICA dissection.

Based on the findings, the patient was diagnosed with compressive neuropathy of the HN caused by dilatation of the ICA loop. He was commenced on acetylsalicylic acid of 300 mg 1x1 to prevent dissection-related recurrent thrombotic and vascular complications. On the follow-up at three months and at one year, the patient’s clinical and imaging findings were stable.

## Discussion

The most common causes of isolated HN palsy are malignancy, surgery or iatrogenic, and idiopathic [1-4]. On the other hand, various arterial or venous vascular causes are rare, as demonstrated in multiple case reports and reported by very few literature reviews [1,2]. These included carotid artery dissection, VA compression, PICA aneurysm, enlarged emissary vein, dural AV fistula, and persistent hypoglossal artery cases. Among the vascular causes of isolated HN palsy identified from studies harboring large numbers of cases, ICA dissections were the most common [1]. VA compression leading to isolated HN palsy was dominantly caused by atherosclerotic vessels [2].

**Figure 4:**
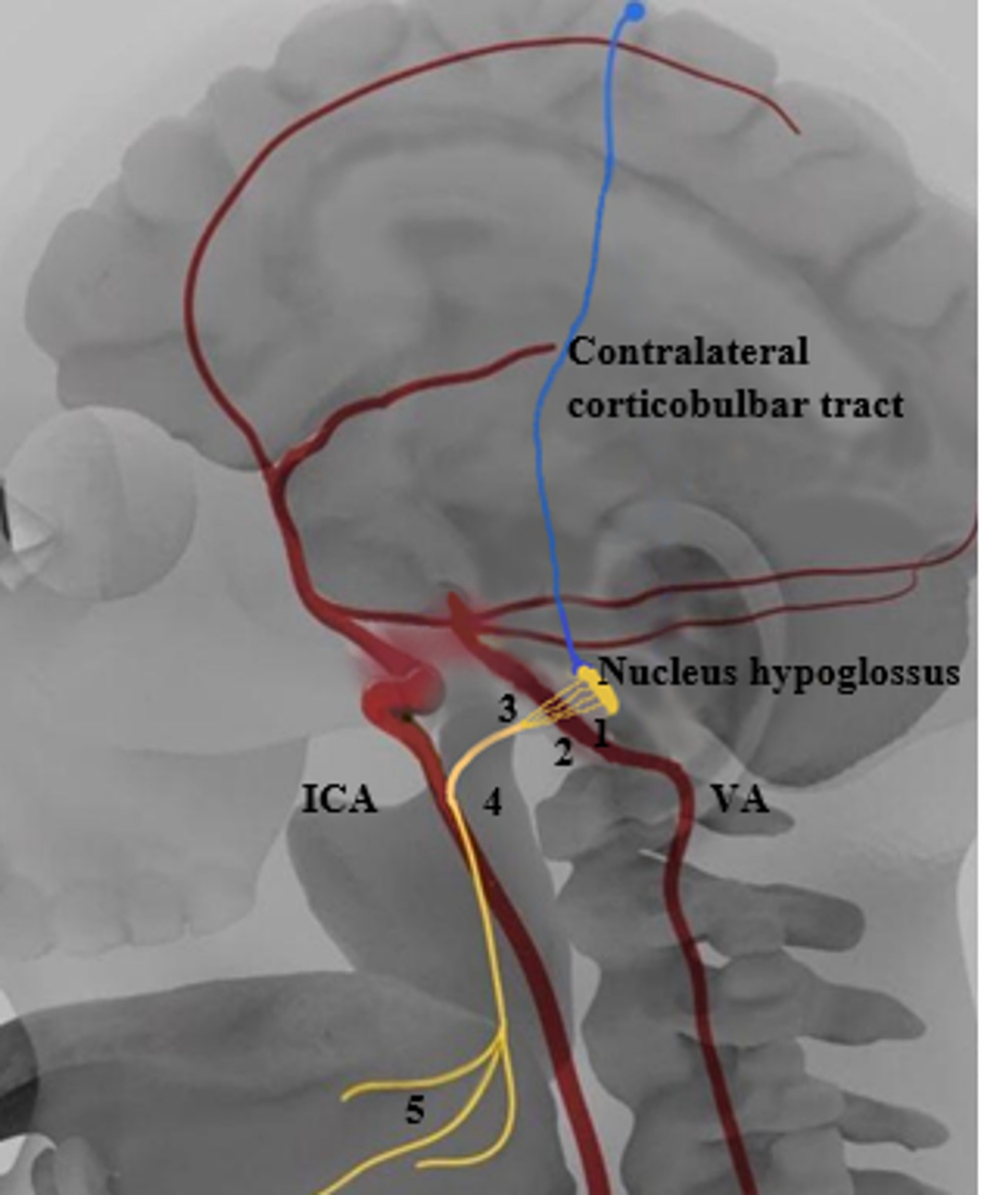
Anatomic relationship of internal carotid artery (ICA) and vertebral artery (VA) with hypoglossal nerve (HN) 1: HN perimedullary segment, 2: HN cisternal segment, 3: HN hypoglossal canal segment, 4: HN carotid space segment, 5: HN sublingual segment.

The HN is prone to be damaged anywhere starting from the motor cortex down to the tongue base (Figure 4). It possesses a pure somatic motor function and its distribution is highly complex. Supranuclear lesions occur at the cerebral cortex, corticobulbar tracts, cerebral peduncles, or pons; they are typically mild asymptomatic and do not cause atrophy because of decussation and ipsilateral innervation [5]. Medullary nucleus is composed of four distinct columns on the floor of the fourth ventricle; nuclear lesions are typically bilateral and coexist with impairment of other lower cranial nerves due to the proximity to other nuclei [6]. Infranuclear segment of the HN up to the tongue can be divided into different segments which may be affected by many different pathological processes including compressive ones within these regions [7]. In the cisternal segment, multiple small rootlets arising from the medulla combine to form HN are closely related to VA and may be affected by their pathology [2]. HN is immediately adjacent to the carotid artery in the carotid space. Because of the location of the ICA in the carotid space, vessel enlargement may lead to dysfunction of the lower cranial nerves [1,8]. While hypoglossal segment is relatively sheltered from major vessels, it might be affected by redundant vascular structures such as dural AV fistula, enlarged emissary vein, persistent hypoglossal artery, or by a loop as in our case.

Similar to other cranial nerves, a complex vascular plexus supplies blood to the HN through tributaries from the external carotid and vertebrobasilar circulation that runs parallel to the fibers. Not only compression of the nerve through the effect of the impinging mass but also synergistic effect of ischemia caused by impairment of the vessels supplying the nerve could contribute to neuropathy [8]. Although it is described in the literature, unilateral isolated palsy caused by distal embolization of the vasa nervorum of the HN is unlikely to originate from the ICA.

As the patient was asymptomatic before the incident and gradually improved after the incident, we believe that the dissection in the coiling segment of the distal cervical ICA and resultant over dilatation of the loop might acutely impair the function of the HN through a local mass effect in our case. It is well known the hematoma formation between vessel wall layers of the carotid artery may cause compression of high cervical pericardial sympathetic fibers and HN [1]. Hematoma may expand toward the adventitia to cause pseudoaneurysm that leads to isolated HN palsy [8]. The relationship between dissections and ICA redundancy has been observed and is well-known by many interventionalists. A recent study confirmed the association between the ICA dissection and coiling as statistically significant, whereas no statistically significant difference was found with arterial vessel elongation [9]. Some prior studies reported mechanical compression of HN due to redundant arterial or venous vascular structures including dolicho VA, PICA aneurysm, and even displaced but non-pathologic VA [2,8,10]. However, the coexistence of ICA coiling and isolated compressive cranial nerve palsy was not published before.

In addition to the classical findings of dual lumen, string sign, and intimal flap, in carotid artery dissection, delicate wall irregularities might be observed especially in the healing phase as in our case [11]. While MR imaging may detect some features of dissection with high sensitivity and specificity, DSA is still the gold standard method for diagnosis [1,11]. We believe that DSA is ancillary in case of an elusive lesion or a complex vascular loop. CT or MR imaging has the advantage of demonstrating the anatomy of hypoglossal canal and surrounding structures. Unfortunately, the MR imaging in our case was missing at the beginning of symptoms. Therefore, we could not interpret the vessel wall for hematoma that might support the diagnosis of dissection.

The standard medical management of dissection includes 3-6 months of administration of an oral anticoagulant or an antiplatelet treatment after heparinization, and follow-up should include intermittent DSA or noninvasive imaging [1,11]. In the presence of existing vascular pathology, as in our case, we believe that lifetime medical treatment and follow-up should be warranted.

The contribution of vascular compression in the pathophysiology of cranial neuralgia is noted in the early surgical literature that identifies patients who benefit from microvascular decompression. Surgery may serve as a valuable option when vascular compression of the HN site is unambiguously identified [2]. Endovascular treatments such as coiling with or without stents in such cases might be successful through remodeling of the abnormally dilated vessel or pathologic vessel wall [8]. In fact, many of the pseudoaneurysms of the dissected ICAs were noted on the coiling segment [9]. Due to this high incidence, the importance of close follow-up and surgical or endovascular treatment whenever needed should be stressed in such cases.

## Conclusions

HN palsy may originate from a number of pathologies occuring anywhere in relation to the central or peripheral nervous system. Among these entities, vascular pathologies are rare and generally cause compressive HN neuropathy that could be identified with meticulous imaging. We present here the first case of an isolated HN palsy, related to ICA loop, in the literature. MRA not only helped to diagnose the vessel coiling, but also disclose the pinch of the HN in the outlet portion of hypoglossal canal. Although DSA is accepted as gold standard in such patients, it can be replaced by MRA as a non-invasive method in the diagnosis and in the long-term follow-up.
